# Supplementary material for: Gene regulation gravitates toward either addition or multiplication when combining the effects of two signals
Source: eLife. 2020 Dec 7;9:e59388. doi: 10.7554/eLife.59388 (PMC7771960; doi:10.7554/eLife.59388)
Supplement: Supplementary file 1. [file elife-59388-supp1.docx]

**Supplemental Table 1. Post-sequencing ATAC-seq metrics for each sample.**

| **Condition** | **Replicate** | **Initial number of read pairs (before alignment)** | **Final number of read pairs (aligned, filtered, duplicates removed)** | **Estimated library size from Picard MarkDuplicates report** | **PCR bottleneck coefficient** | **Percent mitochondrial reads** | **Percent of reads within 500 bp of a RefSeq transcription start site** |
| --- | --- | --- | --- | --- | --- | --- | --- |
| EtOH-control | rep1 | 47,581,870 | 39,444,496 | 262,895,599 | 0.94 | 2.8% | 11.9% |
| EtOH-control-halfDensity | rep1 | 41,086,622 | 34,396,506 | 250,581,572 | 0.95 | 2.4% | 11.5% |
| EtOH-control-highDensity | rep1 | 44,072,790 | 36,117,206 | 231,669,308 | 0.95 | 4.5% | 11.6% |
| RA-low-dose | rep1 | 41,260,701 | 33,591,663 | 217,146,943 | 0.95 | 5.3% | 10.7% |
| RA-med-dose | rep1 | 31,647,052 | 26,123,190 | 179,897,280 | 0.95 | 5.8% | 11.0% |
| RA-high-dose | rep1 | 42,261,199 | 33,811,647 | 199,969,660 | 0.95 | 7.6% | 11.4% |
| TGF-β-low-dose | rep1 | 37,747,257 | 31,026,429 | 202,820,143 | 0.94 | 3.9% | 10.5% |
| TGF-β-med-dose | rep1 | 44,624,493 | 36,419,357 | 224,406,039 | 0.94 | 4.3% | 10.2% |
| TGF-β-high-dose | rep1 | 38,094,133 | 31,396,736 | 208,404,962 | 0.95 | 4.8% | 10.9% |
| Both-low-dose | rep1 | 52,387,369 | 40,479,658 | 211,802,845 | 0.95 | 10.1% | 10.5% |
| Both-med-dose | rep1 | 49,853,358 | 39,318,933 | 225,987,476 | 0.95 | 8.9% | 10.5% |
| Both-high-dose | rep1 | 42,206,009 | 33,786,922 | 204,244,183 | 0.95 | 8.1% | 10.2% |
| EtOH-control | rep2 | 38,083,282 | 31,779,309 | 226,734,741 | 0.94 | 2.5% | 11.5% |
| EtOH-control-halfDensity | rep2 | 43,361,513 | 36,593,932 | 269,862,386 | 0.94 | 1.5% | 10.6% |
| EtOH-control-highDensity | rep2 | 39,698,228 | 33,195,307 | 255,254,502 | 0.95 | 1.8% | 11.2% |
| RA-low-dose | rep2 | 36,715,104 | 30,292,869 | 207,792,170 | 0.95 | 4.6% | 10.8% |
| RA-med-dose | rep2 | 40,558,424 | 33,800,414 | 250,171,599 | 0.95 | 3.7% | 10.4% |
| RA-high-dose | rep2 | 41,414,691 | 34,358,578 | 244,841,654 | 0.95 | 4.0% | 10.7% |
| TGF-β-low-dose | rep2 | 44,817,804 | 36,658,961 | 215,298,493 | 0.93 | 3.4% | 10.3% |
| TGF-β-med-dose | rep2 | 39,929,259 | 33,087,956 | 238,242,632 | 0.95 | 3.5% | 10.2% |
| TGF-β-high-dose | rep2 | 46,205,287 | 38,202,058 | 251,952,977 | 0.94 | 2.8% | 10.8% |
| Both-low-dose | rep2 | 40,462,498 | 33,185,265 | 250,065,164 | 0.96 | 5.1% | 8.8% |
| Both-med-dose | rep2 | 44,699,468 | 35,917,746 | 219,591,584 | 0.95 | 7.7% | 9.8% |
| Both-high-dose | rep2 | 48,925,327 | 39,315,827 | 229,690,180 | 0.94 | 6.1% | 10.9% |
| EtOH-control | rep3 | 42,812,546 | 35,309,638 | 231,408,229 | 0.94 | 3.2% | 12.3% |
| EtOH-control-halfDensity | rep3 | 41,813,530 | 34,814,520 | 258,011,226 | 0.95 | 3.0% | 11.6% |
| EtOH-control-highDensity | rep3 | 40,546,148 | 33,264,452 | 204,352,162 | 0.94 | 4.1% | 12.5% |
| RA-low-dose | rep3 | 32,762,330 | 26,916,331 | 179,857,516 | 0.95 | 6.1% | 11.4% |
| RA-med-dose | rep3 | 50,878,299 | 40,363,941 | 221,908,252 | 0.94 | 7.4% | 11.4% |
| RA-high-dose | rep3 | 44,212,167 | 33,875,013 | 165,272,130 | 0.94 | 10.7% | 11.7% |
| TGF-β-low-dose | rep3 | 45,587,448 | 37,129,061 | 231,608,100 | 0.94 | 4.7% | 11.3% |
| TGF-β-med-dose | rep3 | 38,493,959 | 31,108,095 | 182,114,358 | 0.94 | 5.7% | 11.1% |
| TGF-β-high-dose | rep3 | 30,124,631 | 24,795,889 | 163,300,521 | 0.94 | 4.7% | 11.4% |
| Both-low-dose | rep3 | 50,426,925 | 38,748,904 | 196,832,887 | 0.94 | 10.3% | 10.9% |
| Both-med-dose | rep3 | 44,423,642 | 34,365,274 | 183,838,974 | 0.95 | 11.0% | 10.8% |
| Both-high-dose | rep3 | 42,164,691 | 32,326,317 | 169,428,244 | 0.95 | 11.2% | 10.6% |
