# Supplementary material for: Gene regulation gravitates toward either addition or multiplication when combining the effects of two signals
Source: eLife. 2020 Dec 7;9:e59388. doi: 10.7554/eLife.59388 (PMC7771960; doi:10.7554/eLife.59388)
Supplement: Transparent reporting form [file elife-59388-transrepform.docx]

***eLife’s* transparent reporting form**

We encourage authors to provide detailed information *within their submission* to facilitate the interpretation and replication of experiments. Authors can upload supporting documentation to indicate the use of appropriate reporting guidelines for health-related research (see [EQUATOR Network](http://www.equator-network.org/%20)), life science research (see the [BioSharing Information Resource](https://biosharing.org/)), or the [ARRIVE guidelines](http://www.plosbiology.org/article/info:doi/10.1371/journal.pbio.1000412) for reporting work involving animal research. Where applicable, authors should refer to any relevant reporting standards documents in this form.

If you have any questions, please consult our Journal Policies and/or contact us: [editorial@elifesciences.org](mailto:editorial@elifesciences.org).

**Sample-size estimation**

- You should state whether an appropriate sample size was computed when the study was being designed
- You should state the statistical method of sample size computation and any required assumptions
- If no explicit power analysis was used, you should describe how you decided what sample (replicate) size (number) to use

Please outline where this information can be found within the submission (e.g., sections or figure legends), or explain why this information doesn’t apply to your submission:

We did not perform an explicit power calculation for gene expression and chromatin accessibility effects prior to performing our study, as there were a wide range of plausible effect sizes that would depend on the gene or region of chromatin. We performed three biological replicates for each RNA-seq and ATAC-seq condition. Three is a standard number of biological replicates for transcriptomics studies and is higher than the two replicates typically performed for ATAC-seq studies. We chose to do three ATAC-seq replicates in part based on the findings of the following study, where the authors found that identifying peaks through a simple “majority vote” of three or more biological replicates was superior to using more elaborate statistical methods with only two biological replicates. This paper is referenced in the methods section:

Yang Y, Fear J, Hu J, Haecker I, Zhou L, Renne R, Bloom D, McIntyre LM. 2014. Leveraging biological replicates to improve analysis in ChIP-seq experiments. *Comput Biotechnol* J 9:e201401002.

**Replicates**

- You should report how often each experiment was performed
- You should include a definition of biological versus technical replication
- The data obtained should be provided and sufficient information should be provided to indicate the number of independent biological and/or technical replicates
- If you encountered any outliers, you should describe how these were handled
- Criteria for exclusion/inclusion of data should be clearly stated
- High-throughput sequence data should be uploaded before submission, with a private link for reviewers provided (these are available from both GEO and ArrayExpress)

Please outline where this information can be found within the submission (e.g., sections or figure legends), or explain why this information doesn’t apply to your submission:

We performed three biological replicates for each RNA-seq and ATAC-seq experiments, where we define one biological replicate to be one physical dish of cells. Replicates are shown in figure 1, described at the beginning of Results and several sub-sections of Methods, and shown in Supplementary Table 1. We did not perform technical in addition to biological replicates.

We encountered two outlier samples in our RNA-seq analysis, finding them to have much higher amounts of ribosomal RNAs than the rest of the samples. We discarded these two samples and re-performed both RNA extraction and library preparation, testing the originally extracted RNA and newly extracted RNA. We found normal amounts of ribosomal RNA after sequencing these “technical-rerun” samples and pooled together the new results from the original RNA extraction and second RNA extraction that both underwent a new mRNA selection step in the second RNA-seq library preparation protocol.

Raw and processed RNA and ATAC sequencing data is available in links provided under “Data and code availability”, and is currently also being submitted to NIH GEO.

**Statistical reporting**

- Statistical analysis methods should be described and justified
- Raw data should be presented in figures whenever informative to do so (typically when N per group is less than 10)
- For each experiment, you should identify the statistical tests used, exact values of N, definitions of center, methods of multiple test correction, and dispersion and precision measures (e.g., mean, median, SD, SEM, confidence intervals; and, for the major substantive results, a measure of effect size (e.g., Pearson's r, Cohen's d)
- Report exact p-values wherever possible alongside the summary statistics and 95% confidence intervals. These should be reported for all key questions and not only when the p-value is less than 0.05.

Please outline where this information can be found within the submission (e.g., sections or figure legends), or explain why this information doesn’t apply to your submission:

We describe our general statistical approaches in Methods and describe specific statistical tests used in figure captions.

Raw data is shown in figures 1 and 2, with individual replicates highlighted in figure 1C.

N is clearly labeled for all averaged or summarized results. Exact p values are reported in the Results section whereas “binned” p values are marked with a varying number of asterisks in the figures, with bins defined in the figure caption.

(For large datasets, or papers with a very large number of statistical tests, you may upload a single table file with tests, Ns, etc., with reference to sections in the manuscript.)

**Group allocation**

- Indicate how samples were allocated into experimental groups (in the case of clinical studies, please specify allocation to treatment method); if randomization was used, please also state if restricted randomization was applied
- Indicate if masking was used during group allocation, data collection and/or data analysis

Please outline where this information can be found within the submission (e.g., sections or figure legends), or explain why this information doesn’t apply to your submission:

This information may not be relevant for our submission given that we worked primarily with cell lines.

In case this is relevant: during RNA-extraction, ATAC-seq library preparation, and RNA-seq library preparation, within each biological replicate we assigned each sample a random number between 1 and 36, and used this random number as a sample’s identifier as it proceeded through each experimental step.

**Additional data files (“source data”)**

- We encourage you to upload relevant additional data files, such as numerical data that are represented as a graph in a figure, or as a summary table
- Where provided, these should be in the most useful format, and they can be uploaded as “Source data” files linked to a main figure or table
- Include model definition files including the full list of parameters used
- Include code used for data analysis (e.g., R, MatLab)
- Avoid stating that data files are “available upon request”

Please indicate the figures or tables for which source data files have been provided:

Our public github page: https://github.com/emsanford/combined_responses_paper

Includes all scripts used to generate each figure for the paper (prior to resizing, coloring, and arranging in Adobe Illustrator, and with the exception of raw ATAC-seq tracks that were exported from IGV).

Raw and processed RNA and ATAC sequencing data, including all code and intermediate analysis files, are available in links provided under “Data and code availability” section of the manuscript.
